# ABC- and SLC-Transporters in Murine and Bovine Mammary Epithelium - Effects of Prochloraz

**DOI:** 10.1371/journal.pone.0151904

**Published:** 2016-03-30

**Authors:** Yagmur Yagdiran, Agneta Oskarsson, Christopher H. Knight, Jonas Tallkvist

**Affiliations:** 1 Department of Biomedical Sciences and Veterinary Public Health, Swedish University of Agricultural Sciences, SE-750 07 Uppsala, Sweden; 2 Department of Veterinary Clinical and Animal Sciences, University of Copenhagen, 1870 Frederiksberg C, Denmark; University of Houston, USA, UNITED STATES

## Abstract

Some chemicals are ligands to efflux transporters which may result in high concentrations in milk. Limited knowledge is available on the influence of maternal exposure to chemicals on the expression and function of transporters in the lactating mammary gland. We determined gene expression of ABC and SLC transporters in murine mammary tissue of different gestation and lactation stages, in murine mammary cells (HC11) featuring resting and secreting phenotypes and in bovine mammary tissue and cells (BME-UV). Effects on transporter expression and function of the imidazole fungicide prochloraz, previously reported to influence *BCRP* in mammary cells, was investigated on transporter expression and function in the two cell lines. Transporters studied were BCRP, MDR1, MRP1, OATP1A5/OATP1A2, OCTN1 and OCT1. Gene expressions of *BCRP* and *OCT1* in murine mammary glands were increased during gestation and lactation, whereas *MDR1*, *MRP1*, *OATP1A5* and *OCTN1* were decreased, compared to expressions in virgins. All transporters measured in mammary glands of mice were detected in bovine mammary tissue and in HC11 cells, while only *MDR1* and *MRP1* were detected in BME-UV cells. Prochloraz treatment induced MDR1 gene and protein expression in both differentiated HC11 and BME-UV cells and increased protein function in HC11 cells, resulting in decreased accumulation of the MDR1 substrate digoxin. In conclusion, our results demonstrate that murine (HC11) and bovine (BME-UV) mammary epithelial cells can be applied to characterize expression and function of transporters as well as effects of contaminants on the mammary transporters. An altered expression, induced by a drug or toxic chemical, on any of the transporters expressed in the mammary epithelial cells during lactation may modulate the well-balanced composition of nutrients and/or secretion of contaminants in milk with potential adverse effects on breast-fed infants and dairy consumers.

## Introduction

Milk and dairy products are valuable foods for humans constituting an important nutrient source [1] but may also contain hazardous compounds [2–4]. Chemicals are secreted from blood to milk either via passive diffusion or active transport. Passive diffusion of chemicals can be predicted by taking physical and chemical properties into account [5] and result in levels of hydrophilic compounds in milk equal to or lower than in plasma. However, in cases where active transport mechanisms are operative chemical compounds may be concentrated in milk to a high extent which may pose a health threat to both breast-fed infants and dairy consumers [6, 7].

Transporters are transmembrane proteins involved in cellular in- and efflux of essential and non-essential chemicals. Because these transporters are highly expressed in epithelia of tissues they serve an important role in absorption, tissue distribution and excretion of drugs and other chemicals [8]. Although a vast knowledge has been gained during the last decades concerning transport protein function, localization and expression in many tissues of the body, there is limited data on transporters in the mammary gland and their role for secretion of contaminants into milk [7–12]. In addition, very little attention has been paid to the influence of maternal exposure to chemicals on the expression and function of transporters in the lactating mammary gland.

Expressions of transport proteins belonging to both the ATP-binding cassette (ABC-) and Solute Carrier (SLC-) superfamilies vary with lactation stage of the mammary gland [7, 9, 10]. The transporters play pivotal roles in the delivery of e.g. vitamins, fatty acids, sterols, porphyrins, thyroid hormones, carnitine, and amino acids to the breast-fed offspring [6, 13, 14]. However, some of these transporters feature broad substrate specificities and have been demonstrated to mediate active transport also of drugs and toxic chemicals into milk [7, 9, 14–16]. Transporters with broad substrate specificities and altered expressions in lactating as compared to resting mammary epithelial cells include breast cancer resistance protein (BCRP/ABCG2) [6, 7, 13, 17, 18], multidrug resistance protein 1(MDR1/ABCB1) [7, 18], multidrug resistance-associated protein 1 (MRP1/ABCC1) [7, 10], solute carrier organic anion transporter family member 1A2 (OATP1A2/SLCO1A2), organic cation transporter novel protein type 1 (OCTN1/SLC22A4) [10] and organic cation transporter 1 (OCT1/SLC22A1) [16]

Prochloraz (N-propyl-N-[2-(2,4,6-trichlorophenoxy)ethyl]-1H-imidazole-1-carboxamide), an imidazole fungicide used in agriculture and horticulture [19] is an endocrine disruptor with various modes of action [20, 21]. It has been reported that prochloraz treatment increases *BCRP* expression and efflux activity through AhR activation in primary bovine mammary epithelial cells and in Madin-Darby canine kidney II (MDCK II) cells transfected with caprine *BCRP* cDNA [22, 23].

Cell models are valuable to get information on active transport of chemicals. MDCK II cells and human intestinal epithelial Caco-2 cells are examples of models used to study substrate specificity and function of transporters [24]. In the present study we used non-tumorigenic cell lines derived from mammary tissue, in order to study molecular expression and function of endogenous transporters. HC11 cells are derived from mammary gland tissue of BALB/C mice during gestation and can be differentiated by treatment with lactogenic hormones. After treatment the cells feature a secreting phenotype [25–27]. We have recently demonstrated that BCRP and MDR1 are expressed in HC11 cells and are up- and downregulated, respectively by differentiation of the cells, similar to the situation *in vivo* during lactation [18]. In the present study we also used bovine mammary epithelial BME-UV cells, derived from a lactating Holstein cow, and previously reported to express *MDR1* [28].

The aims of the present study were to (i) investigate gene expressions of endogenous *BCRP*, *MDR1*, *MRP1*, *OATP1A5/OATP1A2*, *OCTN1* and *OCT1* in mammary glands of mice in virgins and at various gestation and lactation stages as well as in mammary glands of lactating cows (ii) characterize gene expressions of the transporters in HC11 and BME-UV cells and (iii) assess the effect of prochloraz on expression and function of transporters in the two cell lines.

## Materials and Methods

### Reagents and chemicals

Roswell Park Memorial Institute (RPMI) 1640 basal medium, gentamicin, heat–inactivated fetal bovine serum (FBS), Ham’s F12, NCTC 135 and 0.05% Trypsin-EDTA, Hank’s Balanced Salt Solution with CaCl_2_ and MgCl_2_ were from Gibco (Invitrogen). Human insulin, epidermal growth factor (EGF), prolactin, hydrocortisone, lactose, lactalbumin hydrolysate, GSH, L-ascorbic acid, prochloraz (N-propyl-N-[2-(2,4,6-trichlorophenoxy)ethyl]-1H-imidazole-1-carboxamide) and N-(2-hydroxyehtyl) piperazine-N’-(2-ethanesulfonic) acid (HEPES) were obtained from Sigma-Aldrich. Nucleospin RNA purification kit was purchased from Macherey-Nagel and Quant-iT™ RiboGreen®RNA Assay Kit from Life Technologies. One-tube QuantiTect™SYBR®Green RT-PCR Kit was obtained from Qiagen and Cell TagTM 700 Stain In-Cell WesternTM Assay Kit was purchased from Li-Cor. Digoxin was kindly provided by Dr. Per Artursson, Department of Pharmacy, Uppsala University, Sweden and ^3^H-digoxin purchased from PerkinElmer.

### Mammary gland isolation from mouse and cow

NMRI-mice were given a standard pellet diet and tap water ad libitum under standard conditions of temperature and light. Animals were killed by cervical dislocation and mammary glands from virgin, pregnant (gestation days 13 and 18), lactating (lactation days 2 and 9) and weaning (weaning day 2) mice were rapidly excised, placed in RNAlater (Invitrogen), snap-frozen in liquid nitrogen and stored at -70°C pending isolation of total RNA. Animal experiments were reviewed and approved by the Council for Animal Experimentation of the Danish Ministry of Food, Agriculture and Fisheries at the Danish Veterinary and Food Administration (permit no. 2012-15-2934-00587) and conducted at the Danish Technical University, Copenhagen, Denmark.

Mammary gland tissues of lactating cows were collected fresh at the Funbo-Lövsta abattoir located at the Swedish Livestock Research Centre, Uppsala, Sweden, placed in RNAlater and stored at -70°C pending isolation of total RNA.

### Cell culture

The HC11 murine mammary epithelial cell line was a generous gift from Dr. Nancy Hynes (Friedrich Miescher Institute for Biomedical Research, Basel, Switzerland) [29] and used with the permission of Dr. Bernd Groner (Institute for Biomedical Research, Frankfurt, Germany). Cells were cultured in sterile filtered RPMI 1640 medium containing 10% heat-inactivated FBS, 5 mg/L insulin and 10 μg/L EGF and 50 μg/ml gentamycin in polycarbonate flasks at 37°C in 5% CO_2_. Medium was changed routinely every 2 or 3 days and cells subcultured by trypsination every 3 or 4 days. Cells of passage numbers 8–15 were used. To induce lactogenic differentiation of the cells they were seeded at a density of 500,000 cells/well into 6 well plates and cultured to confluency. Six days post-confluency the cells were incubated in medium without EGF for 24 h. Following this EGF depletion step differentiation of the cells was accomplished by culturing the cells for an additional 72 h in serum-free medium containing 1 μg/ml prolactin and 1μM hydrocortisone. Differentiation of the cells was assessed by measuring induction of β-casein *(CSN2)* gene expression as well as examination of cellular morphology as described previously [18].

The BME-UV bovine mammary epithelial cell line deriving from lactating and pregnant Holstein cow was kindly provided by Dr. Bruce Schultz, College of Veterinary Medicine, Kansas State University, USA and cultured as described by [30]. Briefly, BME-UV cells were cultured in sterile filtered RPMI 1640 medium containing 10% heat-inactivated FBS, 40% Ham’s F12, 20% NCTC 135, 0.1% lactose, 0.1% lactalbumin hydrolysate, 1.2 mM GSH, 10 μg/ml L ascorbic acid, 1 μg/ml hydrocortisone, 1 μg/ml insulin and 50 μg/ml gentamycin in polycarbonate flasks at 37°C in 5% CO_2_. In some instances, BME-UV cells were prolactin treated for 72 h as previously described [31].

### Cell viability

MTS reduction test was applied to measure mitochondrial activity in viable cells where the tetrazolium compound is transformed to colored formazan. A total of 17,000 cells/well were seeded into 96-well plates in a volume of 100 μl, cultured and differentiated as described above. Stock solutions of prochloraz were dissolved in dimethyl sulfoxide (DMSO) and added to the basal medium, 1:1000, to achieve the decided concentrations of the chemical in the presence of 0.1% DMSO. Cells were treated for 24 h with prochloraz (vehicle control, 0.1, 1, 10, 30 and 100 μM) in serum-free RPMI 1640 medium. DMSO at 10% was used as a positive control in the MTS test. The cells were exposed to test substances for 24 h and 20 μl CellTiter 96® AQ_ueos_ One Solution Reagent (Promega Corporation) was added to each well, according to manufacturer’s instructions. After 1 hour incubation at 37°C absorbance was measured at 490 nm using a Wallac Victor^2^1420 microplate reader (Perkin-Elmer). Cell viability was assessed by comparing mean absorbance values from prochloraz treated cells and vehicle controls based on six replicates.

### Cell exposure

HC11 and BME-UV cells were seeded in 6-well culture plates and HC11 cells differentiated as described above. After differentiation of the HC11 cells and accomplished confluency of the BME-UV cells an additional 24 h incubation with exposure-medium (basal medium supplemented with prochloraz to a final concentration of 0.1, 1, 10, 30 μM and vehicle control) was performed. After prochloraz exposure, cells were washed with 2.5 mL 1 x PBS and then lysed with RA1 buffer (Macherey-Nagel) for RNA isolation. RA1 lysates were stored at -70°C freezer prior to isolation of RNA and gene expression analyzes as described below.

### Isolation of total RNA and RT-qPCR analysis

Total RNA from tissues and cells was isolated by using the NucleoSpin RNA kit containing DNAse I (Macherey-Nagel) as recommended by the manufacturer. To check the integrity of the RNA the 28S and 18S ribosomal RNA bands were examined by UV-visualization following agarose gel electrophoresis. Quantification of the RNA was performed with the RNA specific Quant-iT RiboGreen protocol (Molecular Probes) as described by the manufacturer.

Gene specific intron spanning primers to murine and bovine *CSN2 (β-casein)*, *BCRP*, *MDR1*, *MRP1*, *OATP1A5/OATP1A2*, *OCTN1* and *OCT1* were designed by the use of University of California Santa Cruz (UCSC) Genome Browser and Primer3 software. The primers were synthetized by Cybergene (Huddinge, Sweden). The sequences of the primers and the accession numbers for the mouse and bovine sequences are given in Table 1.

**Table 1:** Primer sequences used for the RT-qPCR analyses. Abbreviations, m: murine; b: bovine.

Genes and accession numbers	Primer sequences
***mCSN2*** NM_009972	CTTAACCCCACCGTCCAAT (forward) AGCATGATCCAAAGGTGAAA (reverse)
***mBCRP*** NM_011920.3	CGCAGAAGGAGATGTGTT (forward) TTGGATCTTTCCTTGCTGCT (reverse)
***mMDR1*** NM_011076.2	ATTTGGCAAAGCTGGAGAGA (forward) ACCCTGTAGCCCCTTTCACT (reverse)
***mMRP1*** NM_008576.3	CTGTGCTCACGATTGCTCAT (forward) CAGAGACCAGCTCACACCAA (reverse)
***mOAPT1A5*** NM_001267707.1	GCACAGAGAAAAAGCCAAGG (forward) CTCCAGGTATTTGGGCAAGA (reverse)
***mOCTN1*** NM_019687.3	CCTGTTCTGTGTTCCCCTGT (forward) GGTTATGGTGGCAATGTTCC (reverse)
***mOCT1*** NM_009202.5	CAGGTTTGGCCGTAAGCTCT (forward) GCAACATGGATGTATAGTCTGGG (reverse)
***bCSN2*** M16645.1	GTGAGGAACAGCAGCAAACA (forward) AGGGAAGGGCATTTCTTTGT (reverse)
***bBCRP*** EU570105.1	AACGGCATTCCAGAGACAAC (forward) ATGTGGATCCTTCCTTGCAG (reverse)
***bMDR1*** XM_590317.6	GCAACATTCTTCACCGGTTT (forward) TTGTCCTCCAAATGCAATCA (reverse)
***bMRP1*** AB082124.1	CCGTCCCTGTTCAAAGTGTT (forward) TGACGAAGCAGATGTGGAAG (reverse)
***bOATP1A2*** NM_174654.2	GCTTGTCTTGCTGGTTGTGA (forward) CAGGGATGGCAGATAAGGAA (reverse)
***bOCTN1*** NM_001206989.1	TTCTCGGCTCCTTTGTGTCT (forward) GCCACCACGTAGTTGGAGAT (reverse)

Quantitative gene expression was examined by RT-qPCR using a Rotor-Gene 3000 (Corbett Research) by applying the One-tube QuantiTect™SYBR®Green RT-PCR Kit (Qiagen), according to the manufacturer’s recommendations. Murine and bovine lactating mammary tissue, respectively, was used as positive amplification controls and all primer-pairs tested on RNA isolated from these tissues generated specific RT-PCR products with anticipated amplicon sizes and single melting curve peaks. Final primer concentration for all target genes was 0.4 μM and 75 or 150 ng total RNA was used as template in 12.5 μl RT-qPCR reactions. Non-template controls served as blanks and melt curve analysis was performed for each sample to check the specificity of the obtained PCR products. Expressions of target genes were normalized to the geometric average expression of three appropriate reference genes [32]. Murine reference genes were hypoxanthine-guanine phosphoribosyltransferase (*Hprt*), ribosomal protein L13A (*Rpl13a*) and glyceraldehyde 3-phosphate dehydrogenase (*Gapdh*) [33] and bovine reference genes were ubiquitously expressed transcript isoform 2 (*UXT*), ribosomal protein S9 (*RPS9*) and ribosomal protein S15 (*RPS15*) [34]. Relative quantification of mRNA expressions was performed by comparing the quantification cycle (Cq) between the tissues and treatment groups of cells according to the 2^-(deltadeltaCq)^–method [35]. Cq cycle 35 was used as cut-off for limit of detection of gene expression. Fold differences were calculated setting virgins or untreated control cells to one.

### In-Cell Western Assay

HC11 and BME-UV cells were seeded at a density of 17,000 cells/well in 96-well plates. The cells were cultured and differentiated as described above prior to treatment with 30 μM prochloraz. Control cells were treated with only vehicle. After 24 h of treatment cells were washed once with 1X PBS and fixed with 4% formaldehyde (1X PBS) for 20 min. Cells were then treated with permeabilization solution (1X PBS containing 0.1% Triton X-100) 5 times for 5 min. After permeabilization cells were incubated in blocking buffer (Li-Cor) for 90 min at RT. Primary Mdr1 antibody (JSB-1, Abcam) diluted 1:200 in blocking buffer, was added to the wells and incubation was performed for 2.5 h under gentle shaking. Cells were then washed five times with 1X PBS containing 0.1% Tween-20 (PBS-T) and then hybridized for 1 h with a polyclonal secondary antibody (IRDye 800CW anti-mouse IgG (H+L) (Li-Cor) diluted 1:1000 in blocking buffer. Cell Tag 700 (Li-Cor) was used for normalization and added (1:500) to the diluted secondary antibody hybridization solution. For background subtraction some wells were used as controls and only incubated with secondary antibody. Cells were washed five times with PBS-T and plates were scanned with detection at both 700 and 800 nm using an Odyssey instrument (Li-Cor Biosciences) and data were analyzed by applying Odyssey software. In each well, secondary antibody signal from the 800 nm channel was normalized to Cell Tag 700 signal from the 700 nm channel. Fold differences were calculated setting vehicle-treated controls to one. The whole procedure was performed at room temperature.

The JSB-1 antibody is reported to cross react with pyruvate carboxylase (PC) with a MW of 130 kDa [36]. However, cross reactivity was not apparent in the HC11 cells under the conditions used in our experiments (S1 Fig). Furthermore, data from human mammary glands demonstrates that the abundance of PC is very low [37].

### Accumulation studies

HC11 and BME-UV cells were seeded in 12 well plates and cultured and treated as described above. Digoxin (Sigma) was chosen as a marker to examine the function of MDR1 in the cells using ^3^H-digoxin (Perkin-Elmer Life Sciences), with a specific radioactivity of 5 Ci/mmol, as tracer. Cells were rinsed with 2 x 1.5 ml of 37°C Hank’s Balanced Salt Solution with CaCl_2_ and MgCl_2_ (Invitrogen), pH 7.4 containing 25 mM HEPES (HBSS) and then pre-incubated for 30 min at 37° in 1.5 ml HBSS. After pre-incubation the cells were incubated at 37°C for 60 min with 37°C HBSS containing 1–5 μM digoxin and 3000 Bq ^3^H-digoxin/ml as tracer. At the end of the experiment the cells were rinsed with 3 x 1.5 ml ice-cold HBSS and thereafter lysed by adding 1 ml 0.5 M NaOH to each well. The digoxin concentration in the cells was calculated from the radioactivity measurement by β-spectrometry using a 2810 TR Tri-Carb^®^Liquid Scintillation Analyzer (PerkinElmer) and normalized to total protein concentration in each sample measured by applying the BCA-method.

### Statistical analysis

Statistical analyses were performed by using Minitab 16 software. The results were analyzed by Kruskal-Wallis to detect any significant differences among the various treatment groups, followed by Mann-Whitney to examine statistically significant differences between two groups. The level of significance was set at p≤ 0.05.

## Results

### Gene expressions in mouse mammary gland

**Fig 1:**
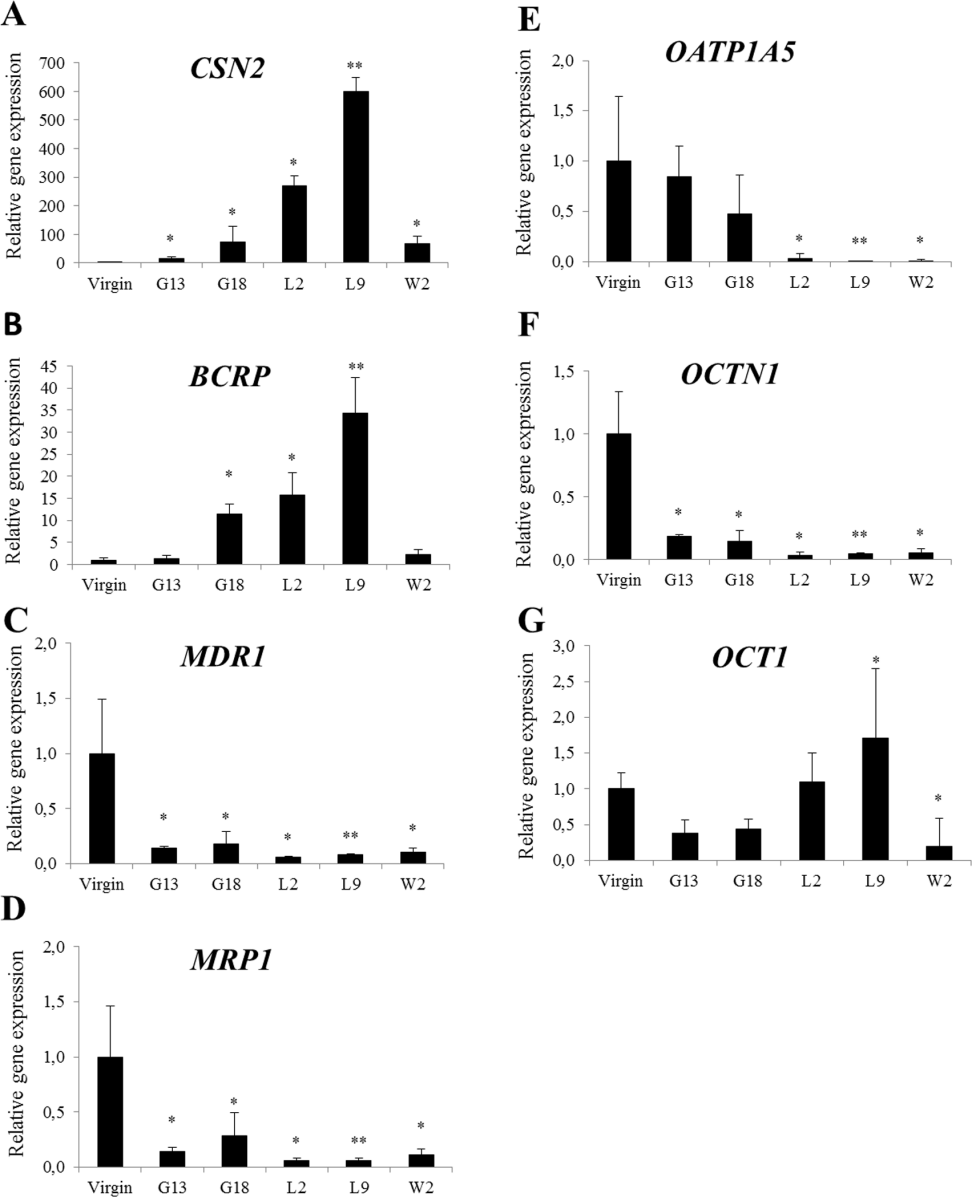
**Relative gene expression of *CSN2* and transporters (A-G) in the mammary gland of mice.** Mammary glands were taken from virgin, pregnant (gestation day 13 and 18), lactating (lactation day 2 and 9) and weaning (weaning day 2) mice. Normalized gene expressions (A-G) are shown relative to virgin and the data is presented as means ± SD; n = 3–4. Statistically significant differences as compared to virgins *p ≤0.05; **p≤0.01.

Messenger RNA expressions of *CSN2*, *BCRP*, *MDR1*, *MRP1*, *OATP1A5*, *OCTN1* and *OCT1* in mammary gland tissues were measured in virgins and at different stages of gestation and lactation from mice. Expressions were normalized to the geometric average of three reference genes and results are presented as relative to virgins (Fig 1). A gradual increase in *CSN2* expression through gestation days 13 and 18 was observed, which continued at lactation day 2 and reached a maximum at day 9 (Fig 1A). At weaning day 2 the gene expression of *CSN2* was reduced to the levels observed at GD 18 (Fig 1A). *BCRP* expression in the mice followed a similar pattern as *CSN2* expression with a continuous increase during gestation and lactation and a relative reduction at weaning day 2 (Fig 1B). In contrast, expression of *MDR1* was decreased through gestation and lactation (Fig 1C). Gene expression of *MRP1* followed a similar pattern as *MDR1* (Fig 1D). *OATP1A5* gene expression was statistically significantly decreased during lactation (Fig 1E). *OCTN1* gene expression had a similar profile as *MDR1* and *MRP1* (Fig 1F). *OCT1* expression was increased at the end of lactation and decreased at weaning day 2 (Fig 1G).

A limited number of cows and without specific data on lactation stages was analyzed. In the bovine mammary gland tissue *CSN2*, *BCRP*, *MDR1*, *MRP1*, *OATP1A2* and *OCTN1* gene expressions were detected (data not shown).

### Gene expressions in cultured mammary epithelial cells

**Fig 2:**
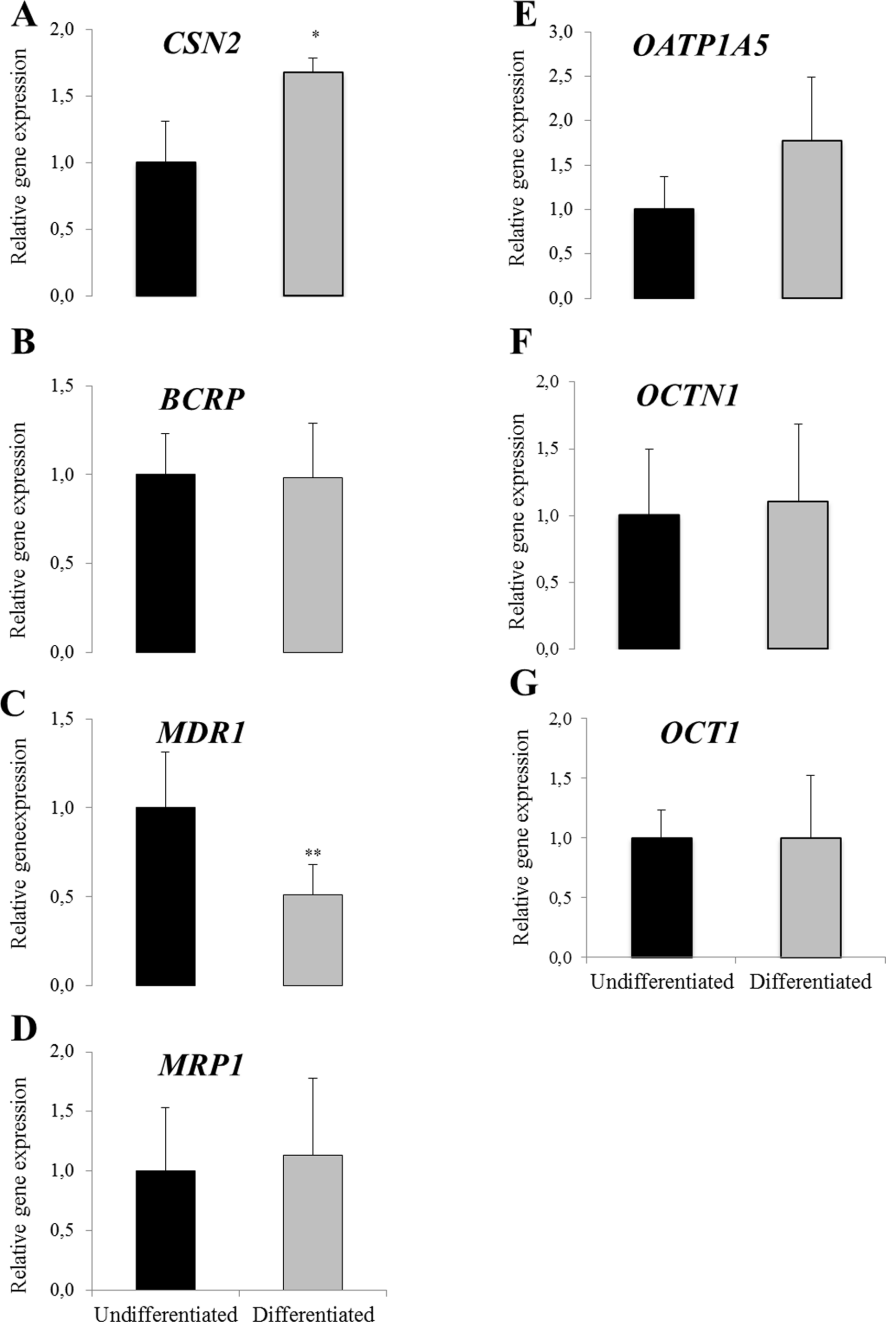
**Relative gene expression of *CSN2* and transporters (A-G) in undifferentiated and differentiated HC11 cells**. Normalized gene expressions (A-G) are presented as means ± SD; n = 6 pooled from 2 separate experiments. Statistically significant differences as compared to undifferentiated controls *p≤0.05; **p≤0.01.

Statistically significant up-regulation in *CSN2* gene expressions was observed in the differentiated HC11 cells as compared to the undifferentiated controls (Fig 2A). *MDR1* gene expression was statistically significantly reduced in the differentiated HC11 cells as compared to the undifferentiated controls (Fig 2C). No statistically significant difference due to differentiation was observed on *BCRP*, *MRP1*, *OATP1A5*, *OCTN1* and *OCT1* gene expressions (Fig 2B, 2D, 2E, 2F and 2G, respectively).

**Fig 3:**
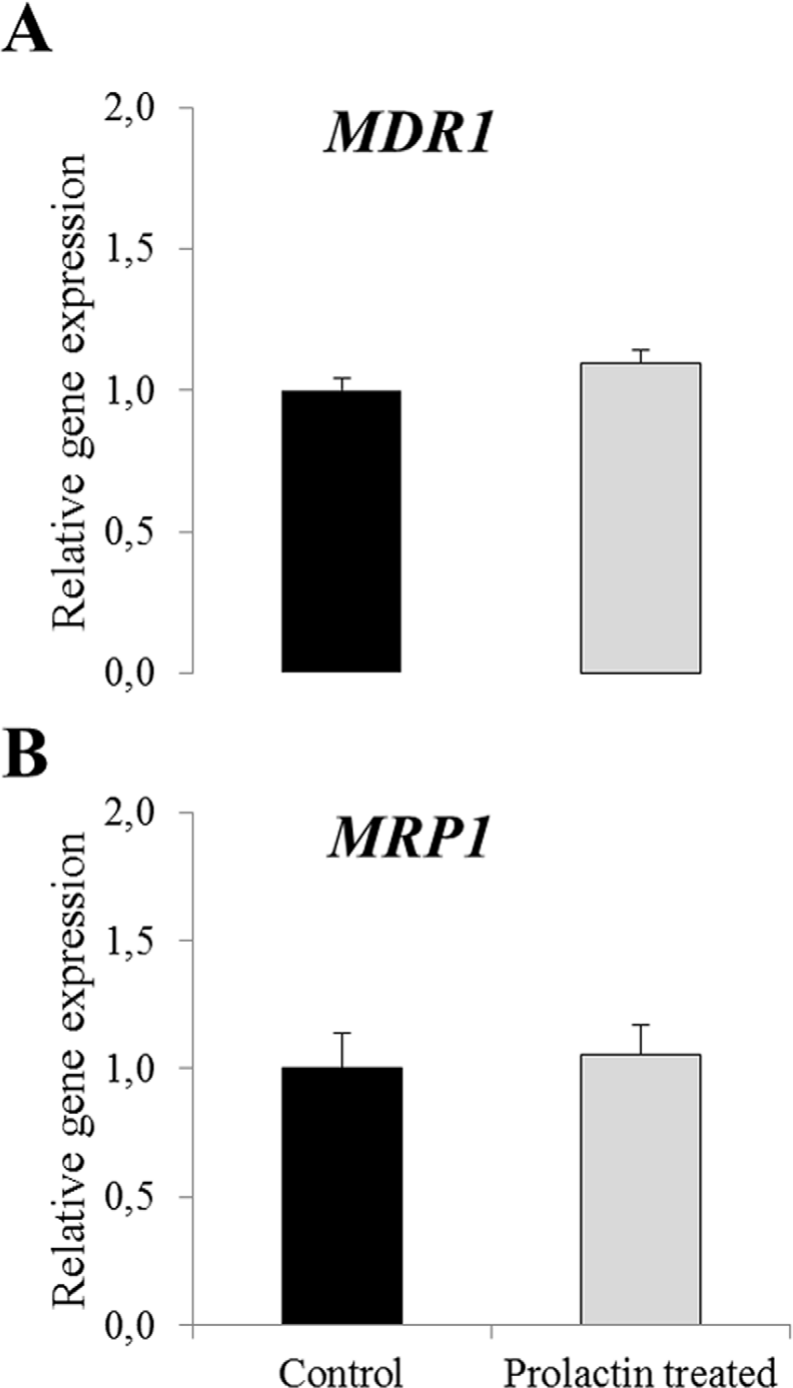
Relative gene expression of transporters in BME-UV cells without (control) and with lactogenic hormone stimulation (prolactin treated). Normalized gene expressions are presented as means ± SD; n = 6). Experiment repeated twice and data shown from one representative experiment.

Among the bovine genes examined only *MDR1* and *MRP1* were detected in the BME-UV cells (Fig 3). No difference was observed in *MDR1* or *MRP1* gene expressions after prolactin treatment of the cells (Fig 3A and 3B).

### Cell viability in prochloraz-treated cells

**Fig 4:**
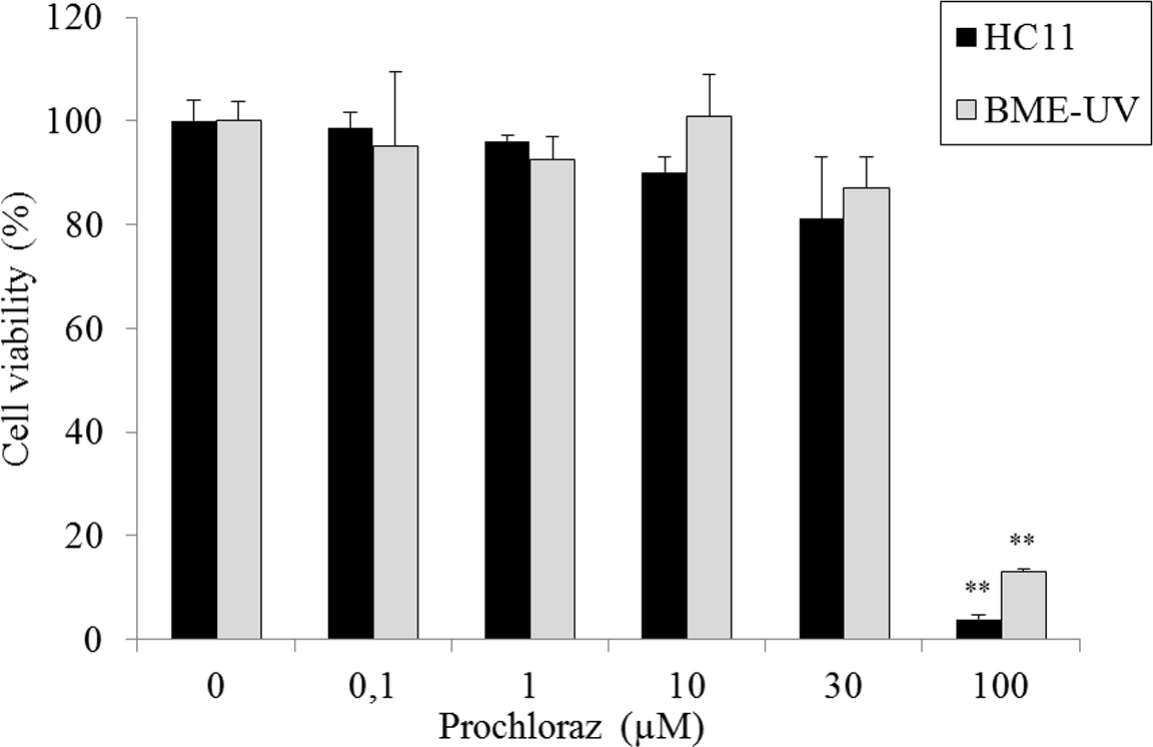
Cell viability in HC11 and BME-UV cells treated with prochloraz for 24h. The data represent means ± SD; n = 6. Statistically significant differences as compared to vehicle controls *p≤0.01. Experiment repeated twice and data shown from one representative experiment.

Cell viability after prochloraz treatment of cells was assessed by MTS. For both cell lines cell viability remained >80% after incubation with prochloraz up to 30 μM compared to vehicle control and declined dramatically after treatment with 100 μM prochloraz (Fig 4).

### Gene expression in prochloraz-treated cells

**Fig 5:**
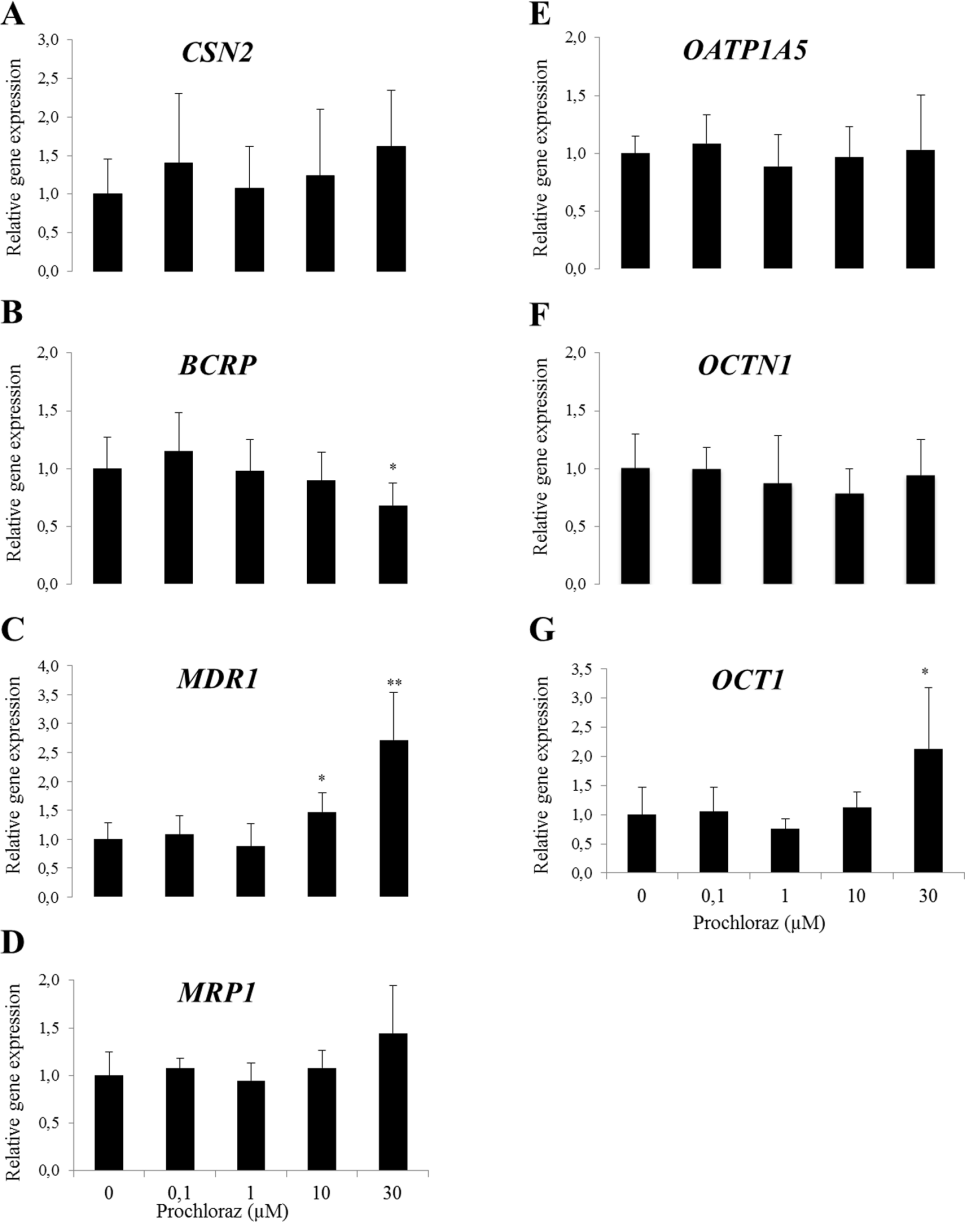
**Relative gene expression of *CSN2* and transporters (A-G) following prochloraz treatment in differentiated HC11 cells.** Normalized gene expressions are presented as means ± SD; n = 6 pooled from 2 separate experiments. Statistically significant differences as compared to vehicle controls *p≤0.05; **p≤0.01.

The effect of prochloraz on gene expression of *CSN2* and transporters was studied in mammary epithelial cells from both cell lines. Prochloraz treatment at non-cytotoxic concentrations resulted in a down regulation of gene expressions of *BCRP* in differentiated HC11 cells (Fig 5B). In contrast, *MDR1* gene expression was significantly up-regulated at the two highest concentrations of prochloraz (10 and 30 μM) (Fig 5C) and OCT1 was significantly up-regulated at the highest concentration (Fig 5G). No statistically significant differences were observed for gene expressions of *CSN2*, *MRP1*, *OATP1A5* and *OCT1* after prochloraz treatment (Fig 5A, 5D, 5E and 5G).

**Fig 6:**
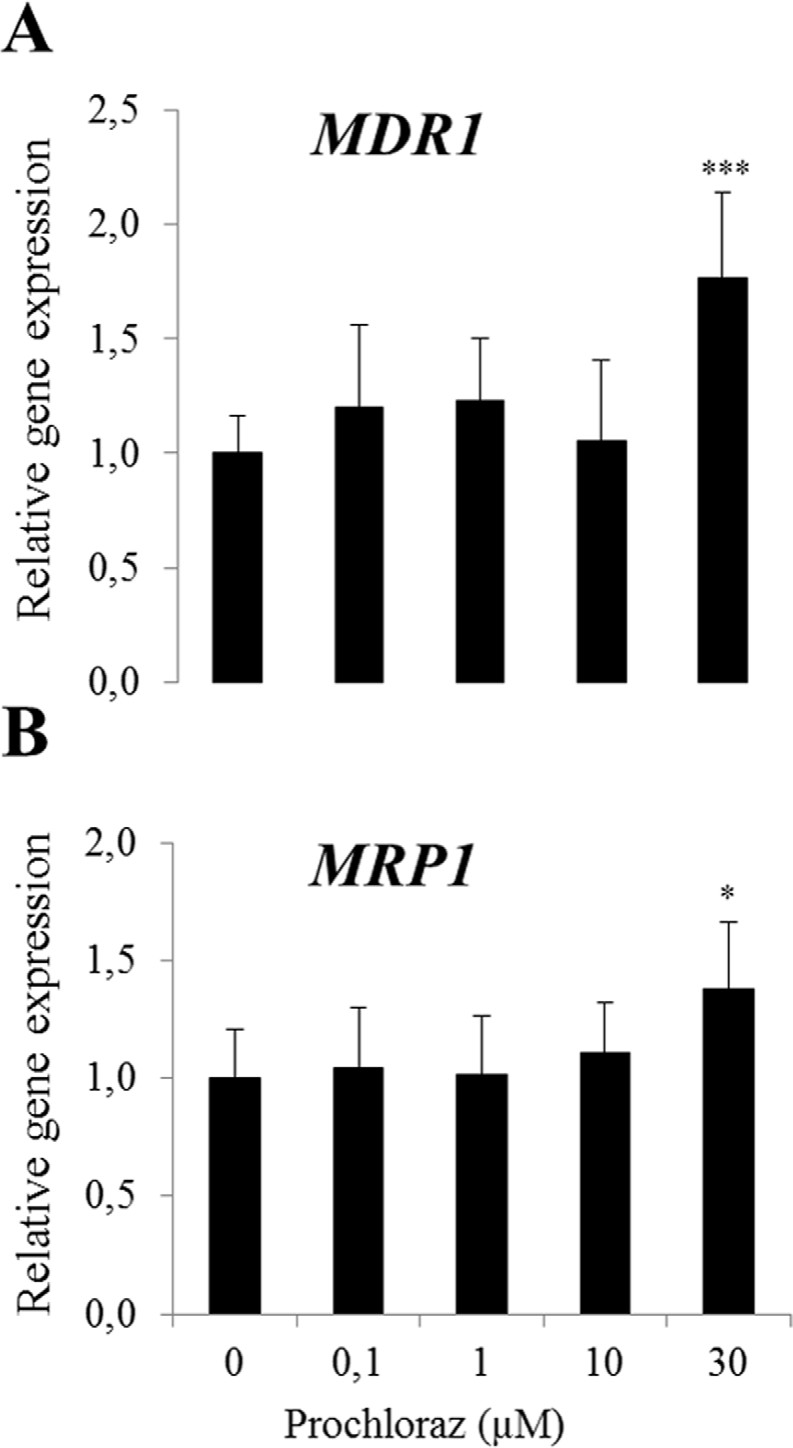
**Relative gene expression of *MDR1* (A) and *MRP1* (B) following prochloraz treatment in BME-UV cells.** Normalized gene expressions are presented as means ± SD; n = 8–12 pooled from 2 separate experiments. Statistically significant differences as compared to vehicle controls, **p≤0.01; ***p≤0.001.

In BME-UV cells, gene expressions of *MDR1* and *MRP1* were induced at the highest concentration of prochloraz (30 μM) (Fig 6A and 6B).

### Protein expression in prochloraz-treated cells

**Fig 7:**
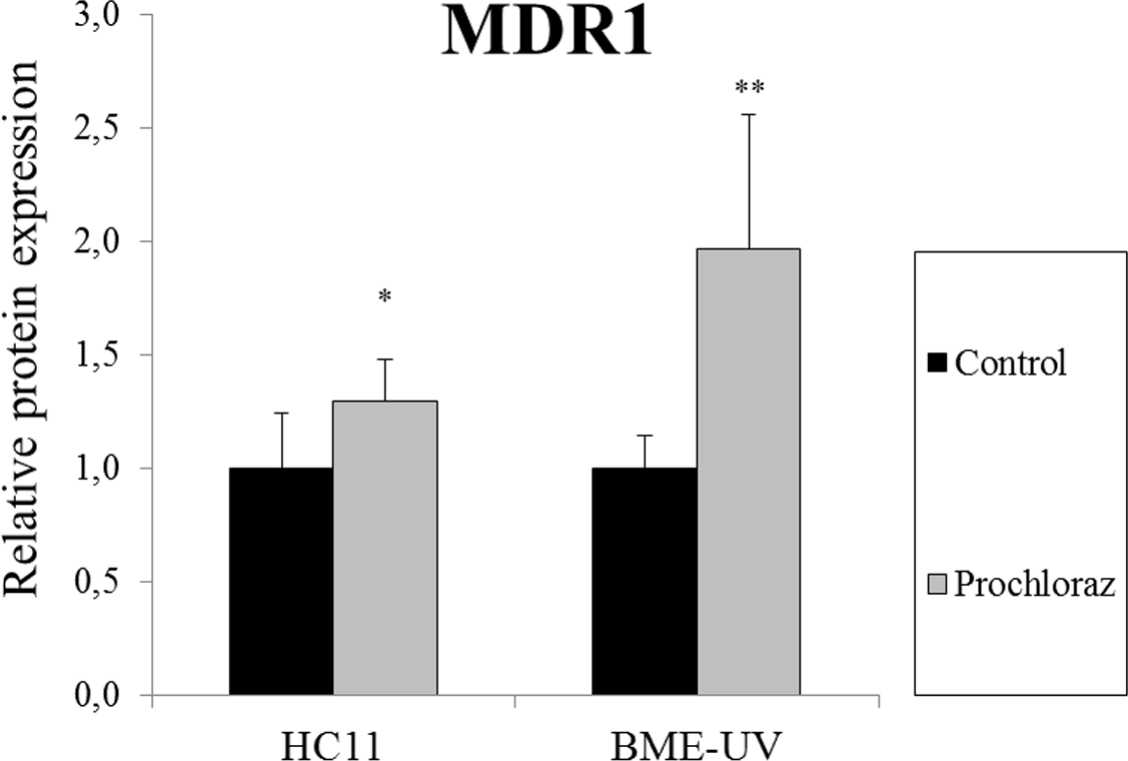
Relative expression of MDR1 following 30 μM prochloraz treatment for 24 h in HC11 and BME-UV cells. The data is presented as means ± SD; n = 4–10. Statistically significant differences as compared to vehicle controls *p≤0.05; **p≤0.01.

Differentiated HC11 and confluent BME-UV cells were exposed to 30 μM prochloraz for 24 h and MDR1 protein expression was examined by an In-Cell Western Assay. MDR1 protein expression was significantly upregulated following 30 μM prochloraz treatment in both HC11 and BME-UV cells (Fig 7).

### Accumulation assay in prochloraz-treated cells

**Fig 8:**
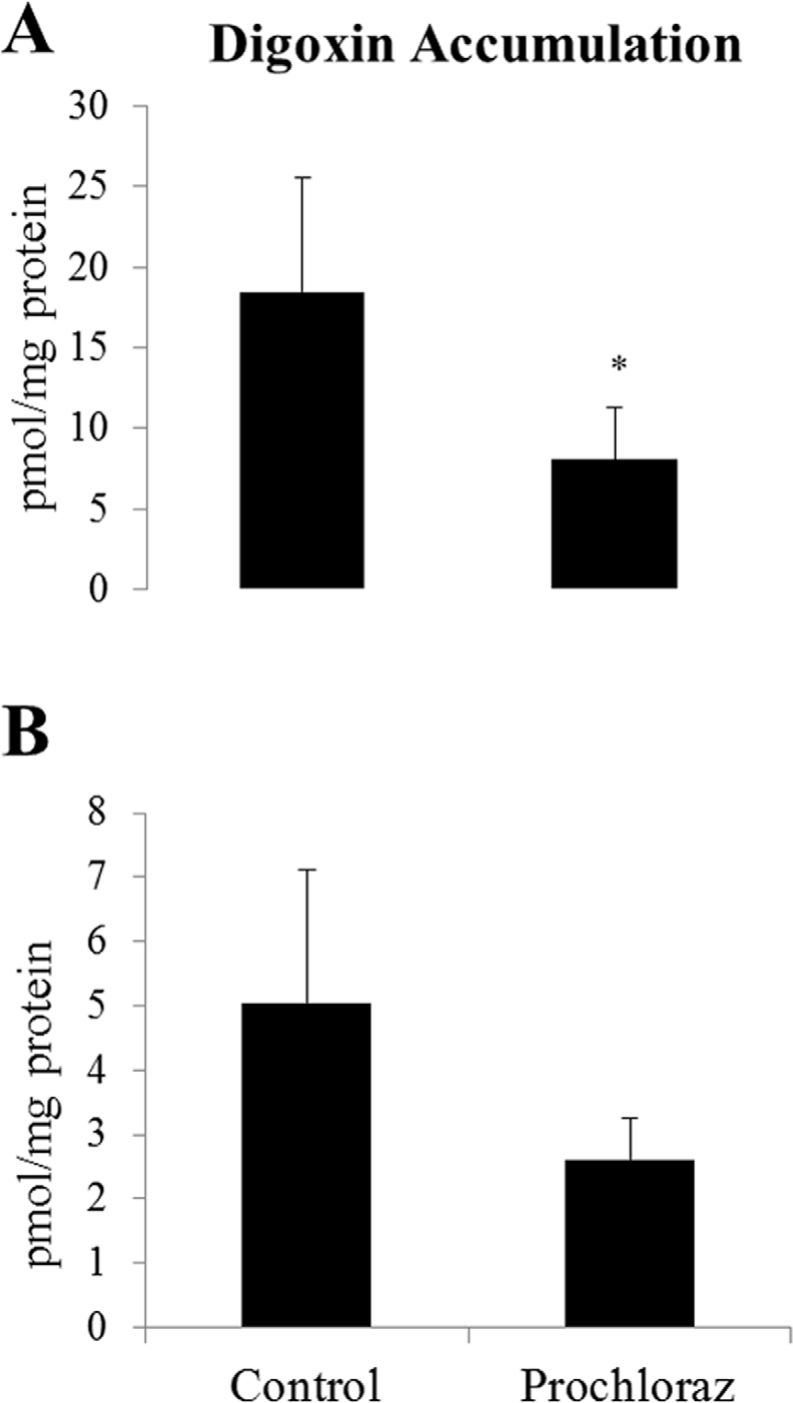
**Accumulation of** ^**3**^**H-digoxin in differentiated HC11 (A) and confluent BME-UV (B) cells, treated with 30** μ**M prochloraz for 24 h.** The data is presented as means ± SD of 3–4 samples and expressed as pmol mitoxantrone/mg cellular protein. Statistically significant differences between groups, *p ≤ 0.05.

Differentiated HC11 and confluent BME-UV cells were exposed to 30 μM prochloraz for 24 h and the function of MDR1 was studied using digoxin as a substrate. Accumulation of digoxin was statistically significantly reduced in prochloraz treated HC11 cells compared to controls cells (Fig 8A). Also in BME-UV cells a lower, although not statistically significant, accumulation of digoxin was observed after prochloraz treatment (Fig 8B).

## Discussion

The expression patterns of ABC- and SLC- transporters in mammary epithelial cells vary during lactation [9, 10, 16]. The function of the transporters in the mammary gland is not known in detail, but includes the delivery of nutrients across the mammary epithelium to the suckling infants via milk. However, some drugs and toxic compounds are ligands to efflux transporters which may result in high concentrations of the chemicals in milk. To identify exogenous substrates for transporters in mammary gland and study the function of transporters, cell models are valuable tools.

We have previously demonstrated gene expressions of *BCRP* and *MDR1* in HC11 cells [18]. In the present investigation we identified gene expressions of *MRP1*, *OATP1A5*, *OCTN1* and *OCT1* in HC11 cells. In the bovine mammary epithelial BME-UV cells only *MDR1* and *MRP1* gene expressions were detected. The lack of expressions of *BCRP*, *OATP1A2* and *OCTN1* in the BME-UV cells may be due to a number of reasons such as loss of transcription factors and selection of cells during passages and experimental conditions [38–40].

In line with previous reports our results demonstrate that both BCRP and OCT1 were most abundant during peak lactation as assessed by the peak in *CSN2* (β-casein) expression, whereas *MDR1* and *MRP1* expressions were reduced at this stage [7, 9, 10, 16, 41, 42]. Stage dependent expression profiles of *BCRP* and *CSN2* were similar, as were the profiles of *MDR1*, *MRP1* and *OCTN1*.

In the present investigation the imidazole fungicide prochloraz was chosen as a test compound to examine the effect on the transporters expressed in the HC11 and BME-UV cell lines. Interestingly, the results showed that prochloraz induced gene and protein expression of MDR1 in both differentiated HC11 and in BME-UV cells. In addition, *MRP1* expression was upregulated by prochloraz in BME-UV cells. Based on predominant expressions of *MDR1* and *MRP1* in excretory organs which have roles in elimination of drugs and xenobiotics, one possible explanation can be that both MDR1 and MRP1 undertake protective roles as efflux pumps in order to prevent accumulation of chemicals and toxic effects in the cells. The apical localization and induction of MDR1 in the apical membranes of mammary epithelial cells may result in an active transport of toxic compounds into milk.

Both gene and protein expressions of MDR1 were increased after treatment of HC11 and BME-UV cells with prochloraz. Our accumulation studies with digoxin, a MDR1 substrate, showed a reduced accumulation in HC11 cells after treatment with prochloraz, indicating an increased function of the efflux transporter MDR1. Also in BME-UV cells treated with prochloraz the accumulation of digoxin tended to be reduced although not statistically significant. The increased MDR1 expression and reduced digoxin accumulation in prochloraz-treated murine mammary epithelial cells emphasizes the risk that chemicals may affect the expression and function of transporters and increase secretion of hazardous chemicals in milk.

The endogenous role of the ABC transporter BCRP has not been clarified, but has been suggested to involve secretion of vitamins including vitamin K3, riboflavin and folic acid into milk [6, 43, 44]. Thus, the induced BCRP expression during lactation in mammary glands may be important for the milk composition in terms of vitamin content. Prochloraz has been reported to increase *BCRP* expression in primary bovine mammary epithelial cells [22] and is transported to milk [45]. However, in the differentiated HC11 cells prochloraz did not induce, but rather reduce *BCRP* expression. Causes for this discrepancy can only be speculated upon but may depend on differences in experimental conditions such as prochloraz concentrations used, exposure times, or species differences in the display of transcription factors and regulation in *BCRP* gene expression. It can be noted that 2,3,7,8-tetrachlorodibenso-*p*-dioxin (TCDD) treatment in the human intestinal C2bbe1 (a subclone of Caco2) cells as well as in other human secondary carcinoma cells of the colon, liver, and mammary glands resulted in induction of *BCRP* transcripts but failed in mouse-derived hepatic (hepa1c1c7), mammary (EMT 6), and intestinal (CMT93) cell lines [46].

In summary, the results demonstrate that murine mammary epithelial HC11 cells featuring a secreting phenotype express endogenous *BCRP*, *MDR1*, *MRP1*, *OATP1A5*, *OCT1* and *OCTN1*. Gene and protein expression of the efflux protein MDR1 is upregulated by prochloraz treatment in both murine HC11 and in bovine mammary epithelial BME-UV cells, which in the HC11 correlates to a decreased accumulation of the MDR1 substrate digoxin. Our findings demonstrate that murine (HC11) mammary epithelial cells can be applied to characterize effects of toxic compounds on expression or function of *BCRP*, *MDR1*, *MRP1*, *OATP1A5*, *OCTN1* and *OCT1*, whereas *MDR1* and *MRP1* can be assessed in bovine (BME-UV) mammary epithelial cells. An altered expression on any of the transporters expressed in the mammary epithelial cells during lactation may modulate the composition of nutrients and/or secretion of contaminants in milk with potential adverse effects on breastfed infants and dairy consumers.

## Supporting Information

S1 Fig Western blot of HC11cell lysates showing distinct MDR1 bands (170 kDa) and only weak bands at other MWs, including 130 kDa, which is the MW of pyruvate carboxylase. Cells were cultured in T75 tissue culture flasks at 37°C in 15 ml sterile filtered RPMI 1640 medium containing L-glutamine and 25 mM HEPES supplemented with 50 μg/ml bovine insulin, 10 ng/ml epidermal growth factor, 7.5% NaHCO_3_ and 10% heat-inactivated fetal bovine serum in an atmosphere of 95% air and 5% CO2 in 95% relative humidity. Cells were harvested by trypzination and homogenized with 5 volumes of RIPA lysis buffer in 1.5 ml Eppendorf tubes by pipetting. Homogenates were incubated on ice for 30 min and then centrifuged at 16,000xg for 30 min at 4°C and supernatant protein concentrations determined by the BCA method. Triplicate samples of 20, 50 and 100 μg of cellular protein were separated on a 10% Tris-Glycine polyacrylamide gel under reducing conditions and blotted to nitrocellulose. Blocking was performed with 5% non-fat dry milk powder in Tris-buffered saline containing 0.05% Tween-20 (TBS-T) overnight at 4°C. Hybridization was then performed with primary MDR1 antibody (JSB1, Abcam) diluted 1:200 in TBS-T. Primary MDR1 antibodies were detected by HRP-conjugated secondary antibodies (ab6728, Abcam) diluted 1:7.500 in TBS-T. HRP was detected by ECL Advance (GE Healthcare) and ChemiDoc instrument (Bio-Rad).
(TIF)
