# Supplementary figures and images for: ABC- and SLC-Transporters in Murine and Bovine Mammary Epithelium - Effects of Prochloraz
Source: PLoS One. 2016 Mar 30;11(3):e0151904. doi: 10.1371/journal.pone.0151904 (PMC4814071; doi:10.1371/journal.pone.0151904)

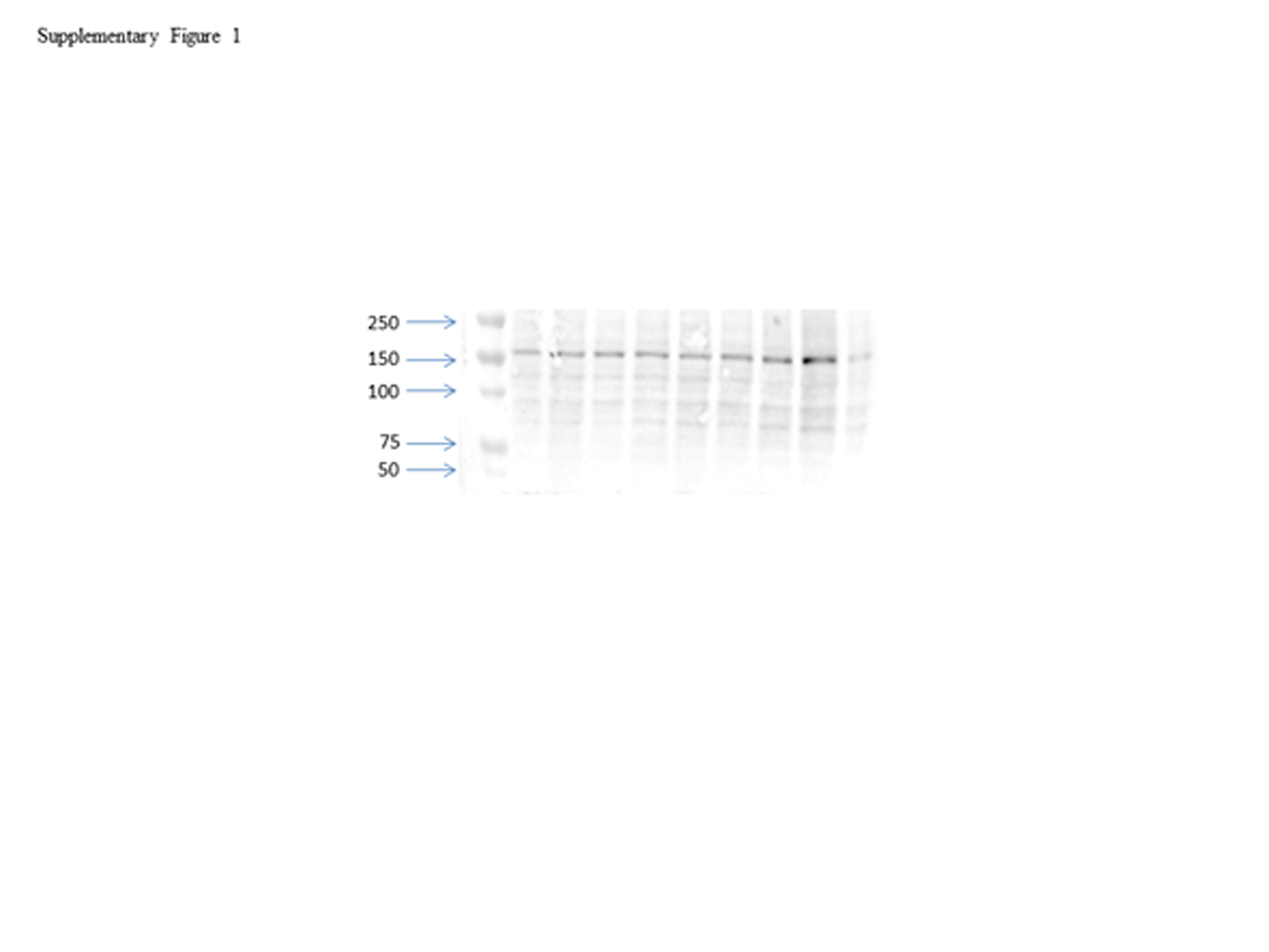

Supplement: S1 Fig — Cells were cultured in T75 tissue culture flasks at 37°C in 15 ml sterile filtered RPMI 1640 medium containing L-glutamine and 25 mM HEPES supplemented with 50 μg/ml bovine insulin, 10 ng/ml epidermal growth factor, 7.5% NaHCO3 and 10% heat-inactivated fetal bovine serum in an atmosphere of 95% air and 5% CO2 in 95% relative humidity. Cells were harvested by trypzination and homogenized with 5 volumes of RIPA lysis buffer in 1.5 ml Eppendorf tubes by pipetting. Homogenates were incubated on ice for 30 min and then centrifuged at 16,000xg for 30 min at 4°C and supernatant protein concentrations determined by the BCA method. Triplicate samples of 20, 50 and 100 μg of cellular protein were separated on a 10% Tris-Glycine polyacrylamide gel under reducing conditions and blotted to nitrocellulose. Blocking was performed with 5% non-fat dry milk powder in Tris-buffered saline containing 0.05% Tween-20 (TBS-T) overnight at 4°C. Hybridization was then performed with primary MDR1 antibody (JSB1, Abcam) diluted 1:200 in TBS-T. Primary MDR1 antibodies were detected by HRP-conjugated secondary antibodies (ab6728, Abcam) diluted 1:7.500 in TBS-T. HRP was detected by ECL Advance (GE Healthcare) and ChemiDoc instrument (Bio-Rad). (TIF) [file pone.0151904.s001.tif]
